# *N*-Methyl-(2S, 4R)-*trans*-4-hydroxy-L-proline, the major bioactive compound from *Sideroxylon obtusifolium*, attenuates pilocarpine-induced injury in cultured astrocytes

**DOI:** 10.1590/1414-431X2022e12381

**Published:** 2022-11-04

**Authors:** P.E.A. Aquino, E.A. de Siqueira, L.C.F. Paes, E.P. Magalhães, T.M. Barbosa, M.A.J. de Carvalho, F.V.C. Serra Azul, I. Rosal Lustosa, M. Mottin, T.L. Sampaio, A.M.C. Martins, E.R. Silveira, G.S.B. Viana

**Affiliations:** 1 Departamento de Fisiologia e Farmacologia, Faculdade de Medicina, Universidade Federal do Ceará, Fortaleza, CE, Brasil; 2 Departamento de Análises Clínicas e Toxicológicas, Faculdade de Farmácia, Odontologia e Enfermagem, Universidade Federal do Ceará, Fortaleza, CE, Brasil; 3 Laboratório de Modelagem Molecular e Design de Fármacos, LabMol, Faculdade de Farmácia, Universidade Federal de Goiás, Goiânia, GO, Brasil; 4 Departamento de Química Orgânica e Inorgânica, Universidade Federal do Ceará, Forteleza, CE, Brasil

**Keywords:** Temporal lobe epilepsy, Voltage-dependent anion channel, Mitochondrial transmembrane potential, Oxidative stress

## Abstract

Glial cells have been implicated in temporal lobe epilepsy in humans and in its models. Astrocytes are lost in several brain regions after acute seizures induced by pilocarpine and may suffer hyperplasia at subsequent time points. This study investigated the effect of *N*-methyl-(2S,4R)-*trans*-4-hydroxy-L-proline (NMP) on astrocytes exposed to cytotoxic concentrations of pilocarpine. Astrocytes were incubated with pilocarpine (half maximal inhibitory concentration (IC_50_)=31.86 mM) for 24 h. Afterwards, they were treated with NMP at concentrations ranging from 3.12 to 100 μg/mL for 24 h. Cell viability was assessed by the 3-(4,5-dimethylthiazol-2-yl)-2,5-diphenyltetrazolium bromide (MTT) assay. Cytoplasmic reactive oxygen species (ROS) and mitochondrial transmembrane potential (ΔΨm) were analyzed by flow cytometry using 2',7'-dichlorofluorescein diacetate (DCFH-DA) and rhodamine-123 (Rho123), respectively. Expression of glial fibrillary acidic protein (GFAP) and voltage-dependent anion channel-1 (VDAC-1) were measured by western blot. Pilocarpine significantly decreased cell viability and mitochondrial potential and increased ROS concentration significantly by 6.7 times compared to the control. NMP concentrations ≥25 µg/mL protected astrocytes against pilocarpine-induced injury in a concentration-dependent manner. Concomitantly, NMP reduced cytoplasmic ROS accumulation to 27.3, 24.8, and 12.3% in the groups treated with 25, 50, and 100 µg/mL NMP, respectively. NMP also protected mitochondria from pilocarpine-induced depolarization. These effects were associated with improvement of pilocarpine-induced GFAP and VDAC-1 overexpression, which are important biomarkers of astrocyte dysfunction. In conclusion, the improvement of ROS accumulation, VDAC-1 overexpression, and mitochondrial depolarization are possible mechanisms of the NMP protective action on reactive astrocytes.

## Introduction

Glia cells perform functions such as nutrition and protection, and help support nervous tissue. Glial cell dysfunction during epileptic seizures is the target of several *in vitro*, *in vivo*, and clinical studies. However, there are still insufficient data to elucidate the precise role of glial activation in epileptogenesis (1). In this context, astrocytes have been studied concerning their role in the pathophysiology of status epilepticus and their importance in the homeostatic balance of the central nervous system. This is because, during status epilepticus, astroglial cells are activated by the presence of cytokines, molecular patterns associated with pathogens, and reactive oxygen species (ROS) (2).

The pathophysiology of seizures is quite complex. Astrocytes respond to all forms of aggression in the central nervous system (CNS) through a process called reactive astrogliosis, a pathognomonic sign of structural damage to this organ (3,4). Concomitantly, astrocytic activation causes the modulation of synaptic activity and neuroplasticity (5). Therefore, significant astrocytic damage contributes to epileptic seizures associated with intense neuronal firing, especially when seizures occur around the nucleus of the lesion (6). Supporting this notion, important CA3 astrogliosis was reported in animals after pilocarpine-induced *status epilepticus* (7).

The glial activation that takes place during a seizure can cause severe and irreversible damage. Although astrocytes can tolerate longer periods of oxygen and glucose deprivation compared to neurons, evidence indicates that alterations in mitochondrial functions in astrocytes can cause an increase in reactive oxygen species and pro-inflammatory markers, causing an imbalance in the local Ca^2+^ signaling. These insults likely lead to a decrease in local production and metabolism of ATP and can induce cell death (8,9).

Therefore, the relationship between glial activation and epileptogenesis needs to be better studied. Some protein and molecular markers are described as being related to astrocytic activation. For example, glial fibrillary acidic protein (GFAP) is a protein expressed by cell types of the extended astroglial family. Although not specific to astrocytes, and often not detectable by immunostaining in nonreactive astrocytes from healthy brains or remote lesions, GFAP has been considered a sensitive and quite specific marker of reactive astrocytes responding to injury in CNS. During a seizure, oxidative stress and astrocytic activation around the central nucleus of the lesion are related to the up-regulation of GFAP (3).

Another widely described marker is the selective channel 1 for stress-dependent anions (VDAC-1), a protein that forms an ion channel in the outer mitochondrial membrane, allowing ATP to diffuse from the mitochondria to the cytoplasm. Within all eukaryotic cells, mitochondria are responsible for the synthesis of ATP among other metabolites necessary for cell survival. VDAC-1, therefore, allows communication between mitochondria and the cell that mediates the balance between cell metabolism and cell death (10).

Recently (11,12), we showed that the methanol fraction isolated from *Sideroxylon obtusifolium* leaves presents a high content of the anticonvulsant bioactive component *N*-methyl-(2S,4R)-*trans*-4-hydroxy-L-proline (NMP). This NMP fraction was shown to increase GFAP and Iba-1 expressions, biomarkers of astrocytes and microglia, in the hippocampus. Further, the anticonvulsive action was demonstrated on the pentylenetetrazole-induced convulsion model in mice, pointing out gamma-aminobutyric acid (GABA) transporter GAT 1 as a target for NMP. Reactive changes in hippocampal astrocytes are frequently associated with temporal lobe epilepsy in humans, as also observed in animal models of seizures (13).

These changes are known to directly influence neuronal excitability and seizure susceptibility (14). Thus, the present study aimed to investigate the neuroprotective effects of NMP by evaluating oxidative stress and hyperactivation of astrocytes, as well as the expression of GFAP and VDAC-1.

## Material and Methods

### Methanol fraction from the leaves of *Sideroxylon obtusifolium*

The bioactive NMP-enriched fraction was obtained by the previously described procedure (15) in which the quantitative ^1^H nuclear magnetic resonance (qHNMR) method was used to determine the NMP concentration. Before all experiments, NMP was dissolved in phosphate buffered saline (PBS).

### Culture of astrocytes

The lineage of murine astrocytes was isolated, immortalized, and assigned by the Federal University of São Paulo (UNIFESP) (16). Cells were cultured in Dulbecco's modified Eagle's medium (DMEM, Invitrogen, USA), supplemented with 10% fetal bovine serum (FBS), 2 mM L-glutamine, and antibiotics (100 U/mL penicillin, 100 μg/mL streptomycin; Sigma-Aldrich, USA) under standard conditions (37°C, 5% CO_2_). Before all experiments, cells were seeded onto 96-well plates or 24-well plates at a density of 1×10^5^ cells/mL overnight to ensure adhesion and proliferation.

### Cell treatment and viability assays

The cell injury protocol used in the present study was based on the treatment of astrocytes with pilocarpine (Sigma-Aldrich) for 24 h, at a toxic concentration for 50% of the cells (IC_50_=31.86 mM), and PBS was used as a negative control. Then, the medium was changed and cells were treated with NMP at decreasing concentrations (100 to 3.12 μg/mL) for 24 h; cells only induced with pilocarpine IC_50_ were used as a positive control.

The cell viability was assessed by the 3-(4,5-dimethylthiazol-2-yl)-2,5-diphenyltetrazolium bromide (MTT, Sigma) assay (17). After treatments, MTT was added to the culture medium at a final concentration of 2.5 mg/mL and incubated at 37°C for 4 h. In viable cells, cytoplasmic and mitochondrial esterases reduce the yellow MTT salt to purple formazan. The resulting formazan was solubilized by adding 10% sodium dodecyl sulfate (SDS) and after 17 h, a spectrophotometric reading was taken at 570 nm.

### Flow cytometry assays

Astrocytes were submitted to flow cytometry analysis to determine mechanisms involved in pilocarpine injury and the protection profile after 24 h exposure to NMP. Cells were displaced using Trypsin-EDTA (0.05%), centrifuged (1500 *g*, 5 min, 4°C), washed twice with binding buffer (10 mM HEPES, 140 mM NaCl, 2.5 mM CaCl2, pH 7.4), and labeled with fluorochromes. The cells were analyzed in a FACS Calibur flow cytometry device (BD Biosciences, USA) using the CellQuest Pro^®^ software (BD Biosciences) to access the production of cytoplasmic reactive oxygen species and mitochondrial transmembrane potential.

Aiming to evaluate cytoplasmic ROS production, the dye 2',7'-dichlorofluorescin diacetate (DCFH-DA, Sigma) was used. This is a lipophilic molecule that freely crosses the plasma membrane and suffers hydrolysis within the cytoplasm, resulting in the formation of a DCFH substrate. Cytosolic ROS oxidize DCFH, forming the fluorescent molecule DCF-Ox, with fluorescent property, exhibiting a green color (18). Astrocytes were labeled with DCFH-DA (100 μM) and analyzed in the flow cytometer for ROS formation, and accumulation was determined by considering the fold change in the geometric mean of FL1 signal intensity relative to the control.

Mitochondrial depolarization was evaluated through the evaluation of the mitochondrial transmembrane potential (ΔΨm) using rhodamine 123 (Rho123) dye. Rho123 is a cationic fluorochrome at physiological pH, which selectively labels mitochondria in living cells. Cells were labeled with Rho123 (10 μg/mL) for 30 min. After that, cells were analyzed in the flow cytometer to measure the decrease in Rho123 accumulation in cell mitochondria. ΔΨm was determined by considering the fold change in the geometric mean of FL2 signal intensity relative to the control (19).

### Expression of GFAP and VDAC by western blot

To extract proteins, astrocytes were washed twice with ice cold PBS. Cells were lysed using 300 μL of RIPA buffer (Tris-HCl 50 mM, pH 7.4, NaCl 150 mM, sodium deoxycholate 0.25%, NaF 10 μg/mL, and EDTA 1 mM) and protease inhibitor cocktail (1:100 phenylmethylsulfonyl fluoride (PMSF) and 2 mM sodium orthovarate). The material contained in six-well plates was mechanically displaced with a cell lifter and centrifuged (15000 *g*, 10 min, 4°C). The quantification of total proteins was performed by the Bronsted colorimetric method (BioRad Laboratories, USA).

Electrophoresis was performed in polyacrylamide gel (PAGE) in a vertical system from BioRad Laboratories mini-PROTEAN^®^ Tetra Cell, as previously standardized (18). A gel composed of 10% polyacrylamide in Tris-HCl buffer 1.5 M, pH 8.8 (BioRad Laboratories) was used as “stacking gel” or “packaging gel” with 5% polyacrylamide in Tris-HCl 0.5 M buffer, pH 6.8. Samples containing 25 μg of the protein were added to wells. Separation was performed using an electrical potential generated by a constant voltage of 180 V and free amperage (PowerPac™ electric power source, HCPower Supply model, USA).

Proteins were transferred to a nitrocellulose membrane (BioRad Laboratories) previously wet in methanol and placed in contact with the gel in appropriate support for electrotransfer by the immersion method (BioRad Laboratories, Model MiniTrans Plot Modulo), with ice transfer buffer (Tris-HCl 25 mM, pH 8.3, glycine 192 mM, and methanol 20%). The electron transference was performed under the difference of electrical potential generated by constant amperage of 400 mA, free voltage, and temperature of 4°C for 2 h.

At the end of the transfer, membranes were blocked overnight with 5% BSA solution in Tris saline buffer containing 0.1% Tween 20 (TBST, pH 8.0) and 1:400 GFAP (∼50 kDa) or 1:2000 VDAC (∼30 kDa) primary antibody diluted in BSA 5% in TBST. The primary anti-actin anti-β (Abcam, UK) was used as an endogenous standard of constitutive protein.

Membranes were washed with TBST and incubated with secondary antibody conjugated with peroxidase enzyme (Abcam) (1:3000, in 5% BSA TBST). For chemiluminescence detection, the Clarity™ ECL (BioRad Laboratories) reagent was used and imaging was performed at chemiDoc™ MP Imaging System (BioRad Laboratories). Image Lab Software™ version 5.1 (BioRad Laboratories) was used for image analysis. After normalization with the expression of the constitutive β-actin protein, a score of 1.0 was assigned to the mean of the control group, and the results of the other groups are reported as units of relative expression.

### Statistical analysis

All data are reported as means±SE. For statistical comparison between the experimental groups, one-way ANOVA was used followed by Tukey's *post hoc* test. As a significance criterion, P<0.05 was accepted. Statistical analyses were performed using GraphPad prism 7.03 software (USA).

## Results

### Cell viability assays

**Figure 1:**
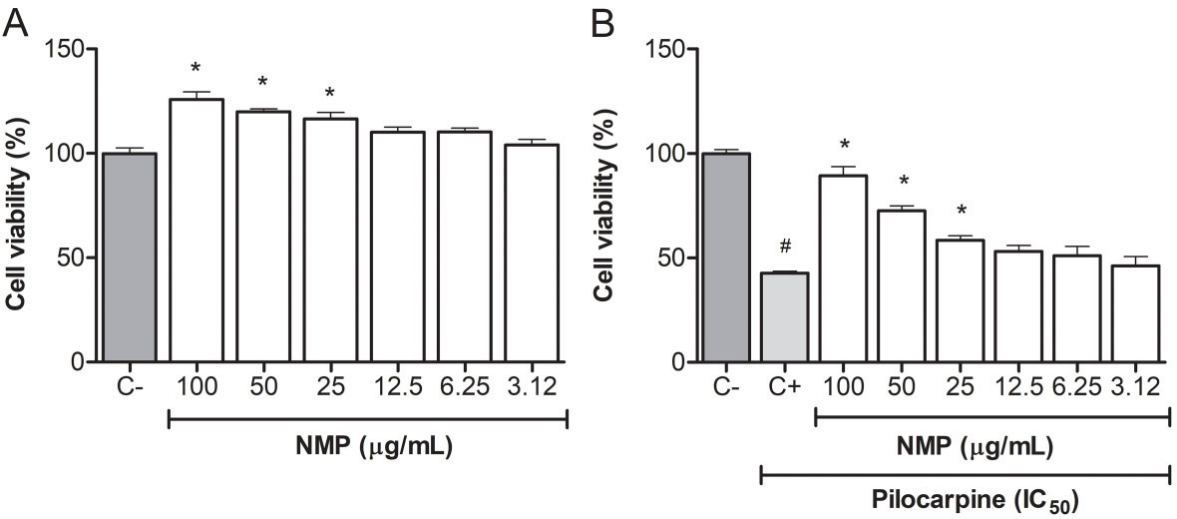
Cell viability of astrocytes treated with NMP (**A**) (F=12.08) or pilocarpine followed by NMP (**B**) (F=42.79). The results are reported as means±SE. *P<0.05 *vs* C-; ^#^P<0.05 *vs* C+ (ANOVA followed by Tukey's *post hoc* test). NMP: *N*-methyl-(2S,4R)-*trans*-4-hydroxy-L-proline; C-: negative control (vehicle); C+: positive control (untreated cells and exposed only to IC_50_ pilocarpine).

Initially, the cells were treated with increasing concentrations of pilocarpine, aiming to obtain the IC_50_, which was equal to 31.86 mM. To assess the cytotoxicity of the methanolic fraction (NMP) on astrocytes, the MTT assay was performed to identify a range of concentrations suitable for work, that is, concentrations that were not toxic. In all concentrations tested, NMP did not show any decrease in cell viability (Figure 1A).

When astrocytes were treated with pilocarpine (IC_50_) for 24 h and subsequently treated with the NMP fraction, it was possible to observe an effect related to the protection of cell viability in concentrations equal to or greater than 25 µg/mL. NMP at the concentrations of 25, 50, and 100 µg/mL produced significant increases in cell viability of 58.3, 72.5, and 89.3%, respectively, compared with the control group (all P<0.05). Then, these three concentrations were chosen for the next experiments (Figure 1B).

### ROS accumulation in astrocytes

To analyze the accumulation of intracytoplasmic ROS in astrocytes, the flow cytometry assay was performed using the DCFH-DA. Pilocarpine increased ROS accumulation by 6.7 times compared to the control (P<0.001). In addition, treatment with NMP reduced ROS accumulation to 27.3% in the group treated with 25 µg/mL, to 24.8% in the 50 µg/mL group, and 1 to 2.3% in the 100 µg/mL group (all P<0.001) compared to the pilocarpine group (Figure 2A).

**Figure:**
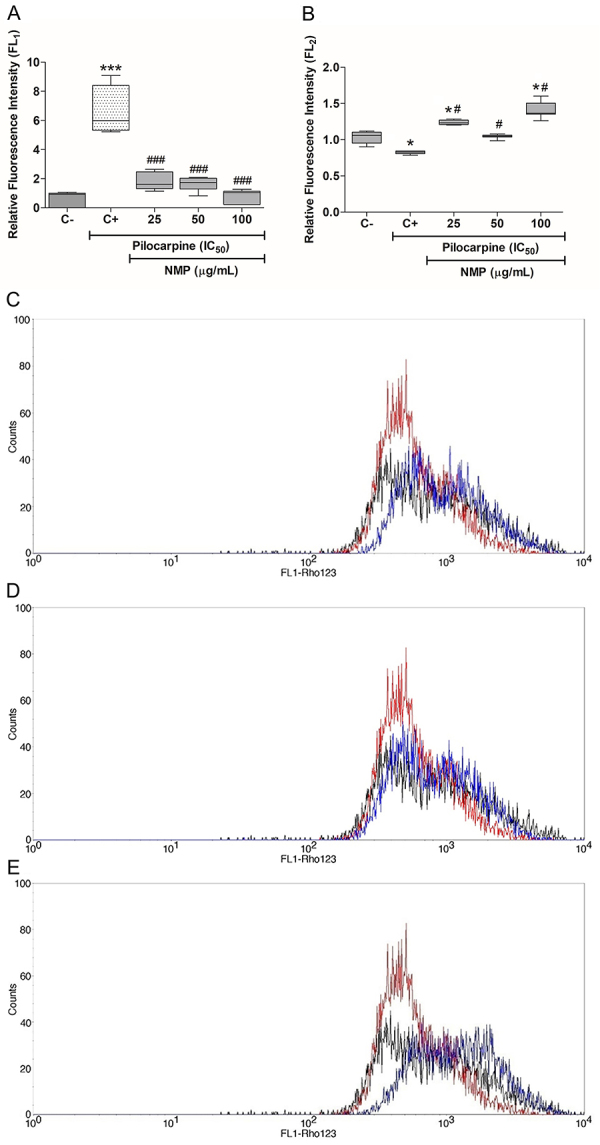
Flow cytometry for analysis of the accumulation of cytoplasmic reactive oxygen species in astrocytes using DCFH-DA assay (**A**) and evaluation of mitochondrial transmembrane potential using rhodamine 123 (**B**). The data are reported as a fluorescence ratio to the ±SE control. *P<0.05, ***P<0.001 *vs* C-; ^#^P<0.05, ^###^P<0.01 *vs* C+ (one-way ANOVA with Tukey’s *post hoc* test). NMP: *N*-methyl-(2S,4R)-*trans*-4-hydroxy-L-proline; C-: negative control (vehicle); C+: positive control (untreated cells and exposed only to pilocarpine at IC_50_). The NMP behavior in different concentrations in potential transmembrane are represented in **C** (25 µg/mL), **D** (50 µg/mL), and **E** (100 µg/mL). The control group and baseline for Δm is represented by the black line, the cell group exposed to IC_50_ pilocarpine-induced injury is indicated by the red line and the peak is shifted to the left, indicating a lower average in fluorescence. The blue line represents the groups of cells exposed to IC_50_ pilocarpine followed by the exposure to NMP. NMP showed a shift of the histogram to the right, demonstrating an approximation to the control group.

### Determination of the mitochondria transmembrane potential

To evaluate the change in mitochondrial transmembrane potential (ΔΨm) caused by pilocarpine and, consequently, by intracellular oxidative stress, a rhodamine 123 flow cytometry test was performed. The results showed that pilocarpine decreased mitochondrial fluorescence by about 20% compared with the control (IFR=1.0), representing 0.83 of the control (P<0.05). The treatment with NMP approached the ΔΨm of the treated groups to that of the control group. Thus, the group treated with NMP 25 µg/mL increased the relative fluorescence intensity by 49.3% compared with the group exposed to pilocarpine (P<0.05), that is, 23% higher than the control value (P<0.05) (Figure 2B). The groups treated with 50 and 100 µg/mL NMP showed IFRs of 1.04 (P<0.05 *vs* the pilocarpine group) and 1.41, respectively (P<0.05 *vs* the control group and *vs* the pilocarpine group), meaning normalization of ΔΨm to levels similar to the control (Figure 2B).

Representative histograms illustrate this behavior, showing the control group (black line) as the baseline for Δm. The group that suffered pilocarpine-induced injury is indicated by the red line, represented by the peak shifted to the left, indicating a lower average of fluorescence. The groups treated with pilocarpine (blue line) followed by exposure to NMP show a shift of the histogram to the right, demonstrating an approximation to the control group (Figure 2C, D, and E).

### Evaluation of GFAP and VDAC-1 expressions in astrocytes cells

To evaluate the influence of pilocarpine and treatments with NMP on the GFAP and VDAC-1 expressions, western blot analyses were performed. We observed that astrocytes submitted to the pilocarpine exposure showed an increased expression of GFAP (7.5-times *vs* control; P<0.05), and this change was reduced by approximately 3 times after exposure to NMP 50 and 100 µg/mL compared with the negative control group (C-) (P<0.05 *vs* the pilocarpine group) (Figure 3A). Although VDAC-1 changes were lower (1.7-times increase in the pilocarpine group relative to control, P<0.05), they were decreased after exposure to NMP 50 and 100 µg/mL to values closer to those of the negative control group (C-) (both P<0.05) (Figure 3B).

**Figure:**
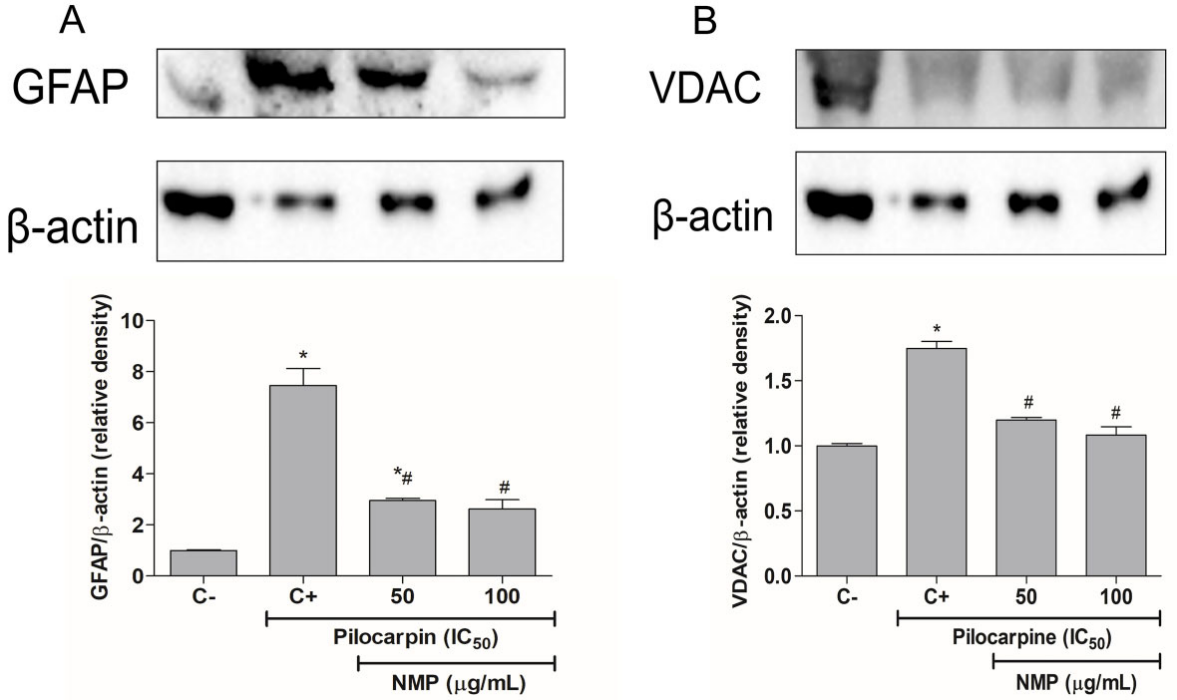
Expression of glial fibrillary acidic protein (GFAP) (**A**) and voltage-dependent anion channel-1 (VDAC) (**B**) in astrocytes challenged with pilocarpine followed by the exposure to NMP. The results are reported as means±SE. *P<0.05 *vs* C-; ^#^P<0.05 *vs* C+ (one-way ANOVA; **A**, F=52.17; **B**, F=57.94), followed by Tukey's *post hoc* test). NMP: *N*-methyl-(2S,4R)-*trans*-4-hydroxy-L-proline; C-: negative control (vehicle); C+: positive control (untreated cells and exposed only to IC_50_ pilocarpine).

## Discussion

The present work showed that NMP protected astrocytes from pilocarpine-induced cell death in a concentration-dependent manner. The protective effect of NMP was related to the improvement of oxidative stress, mitochondrial depolarization, and overexpression of GFAP and VDAC-1, which were parameters found to be affected in concomitance with pilocarpine-induced cell injury.

These results are relevant in view of our recent report of the NMP anticonvulsant action in the intracerebroventricular pilocarpine model in mice (12). In that study, NMP was reported to decrease the pilocarpine-induced neuronal death and overexpression of GFAP and Iba-1 in hippocampal subfields.

Former studies pointed to the antiepileptic action of L-proline and its derivatives (20). The anticonvulsant property of L-proline was demonstrated in pentylenetetrazole-induced seizures in mice, as well as its synergism in potentiating the antiepileptic action of vigabatrin. Thus, a hypothesis of a GABA-mediated mechanism was raised. Nevertheless, the L-proline pharmacodynamics remain to be further investigated.

Pilocarpine-induced seizures *in vivo* are a valuable tool to investigate the mechanisms involved in temporal lobe epilepsy. However, these models need to be standardized in order to reduce mortality from the acute seizures, as well as the large variability in the latent period duration, extension of brain injury, and disease severity regarding the frequency and stage of the spontaneous seizures (21,22). Notwithstanding, pilocarpine-induced seizures in mice show excitotoxic cell death of subsets of hippocampal GABAergic interneurons, particularly in the dentate gyrus and compensatory sprouting of excitatory projections from dentate granule cells, which are also seen in clinical specimens of temporal lobe epilepsy (22,23).

Inflammation and oxidative stress are crosstalk phenomena thought to play a role in seizures and epilepsy. Seizure activity was shown to promote glial activation. Activated glia, in turn, upregulate the production of pro-inflammatory cytokines and their downstream signalizing cascades (24). In particular, astrocytes and myeloid infiltrate were shown to play a main role in seizure-triggered neuroinflammation, while microglia contribute in a lesser extent (25). Thus, modulation of neuroinflammation may make an important frame for therapeutic intervention.

Seizure-induced GFAP overexpression reflects a first step in astrocyte activation and hypertrophy and is significant three days after status epilepticus (14). At this time point, many other proteins are upregulated in astrocytes along with GFAP. One of them, P450 side chain cleavage (P450scc), is an enzyme responsible for the cleavage of the side chain of cholesterol, which is the first step of the neurosteroid synthetic cascade. P450scc has been assigned a role in the modulation of neuronal excitability early after pilocarpine-induced acute seizures (26). Astrocytes can modulate ion channels, receptors, neurotransmitter transporters, and metabolism in accordance with neuronal activity. This is why astrocytes play a crucial role in epilepsy and should be considered as promising targets for new therapeutic strategies (27).

Pilocarpine was shown to kill retinal ganglion cells in culture by an inositol pathway-mediated mechanism, like in other neuron types (28). In cultured corneal stromal cells, pilocarpine was shown to induce apoptosis by a death receptor-mediated mitochondrial-dependent signaling (29).

Different from neurons, calcium transients in astrocytes are mainly triggered by G_q_-protein-coupled receptors and mediated by IP_3_-dependent calcium release from the endoplasmic reticulum store (30). Physiologically, calcium transients are buffered by cytoplasmic proteins and mitochondria before those ions are removed back to interstice and cytoplasmic reticulum to restore the normal low cytoplasmic Ca^+2^ concentration. When the mechanisms responsible for calcium transients are overactivated, for instance during prolonged seizures and excitotoxicity in general, calcium buffering mechanisms are saturated (31). In mitochondria, calcium overload leads to uncoupling of the electron transfer chain, thus making mitochondria an important source of ROS, along with peroxysomes and endoplasmic reticulum (32). A recent study showed that attenuation of inflammation restores calcium-dependent signaling in astrocytes, leading to improvement of memory and learning (33).

In the present work, tests on cultured astrocytes showed that even after cell injury by cytotoxic concentrations of pilocarpine, proline partially protected cell viability. This was concomitant to pronounced decreases in the accumulation of cytoplasmic ROS and mitochondrial depolarization induced by pilocarpine. These data are relevant since oxidative stress is a major contributor to cell death due to oxidation of membrane lipids, functional alteration of proteins and enzymes, and direct damage of nucleic acids (34). Regulation of GSH homeostasis favoring the reduced electrochemical status of the brain tissue improved astrocyte activation and cognitive impairment after pilocarpine-induced status epilepticus (35). Furthermore, the disruption of the electron transfer chain correlated to the drop of transmembrane mitochondrial potential leads to the loss of the ATP synthase activity and finally to the change of the energy threshold of the cell (34).

VDACs are the most abundant proteins in the outer mitochondrial membrane. They are responsible for the transport of metabolites, such as the tricarboxylic acid cycle substrates, between cytoplasm and mitochondria (10,36). Nevertheless, they have been implicated in the apoptosis triggering by mitochondria in neurodegenerative diseases (37). Also, VDAC1 increase and VDAC2 decrease have been documented in epilepsy-related mitochondrial dysfunction (36). NMP protected VDAC1 expression from the significant upregulation seen in pilocarpine-induced cell injury.

L-proline seems to prevent neurodegeneration. A study using glycine-α-methylproline-containing tripeptides has shown that these molecules mitigate neuroinflammation and oxidative stress, as well as the production of nitric oxide by suppression of NF-kB, providing an overall neuroprotective effect (38). Data have emerged supporting the hypothesis that proline may function as a neuromodulator in the brain. L-proline was found to modulate glutamatergic transmission (39). Also, a high affinity proline transporter was detected by immunohistochemistry on subsets of glutamatergic neurons or fibers present in several brain areas, including the arcuate and supraoptic nuclei of the hypothalamus, the trigeminal and magnocellular red nuclei of the mesencephalon, the area postrema and medial longitudinal fasciculus of the medulla, as well as several raphe nuclei through the brainstem (40).

Here, we described the NMP antioxidative action on cytoplasm and membrane lipids, which is associated to protection against mitochondrial depolarization and VDAC-1 and GFAP overexpression in a model of pilocarpine-induced injury in cultured astrocytes. In conclusion, the reduction of ROS accumulation and the improvement of the drop of mitochondrial potential and VDAC-1 overexpression are the probable mechanisms of the anticonvulsant and neuroprotective activities of NMP described elsewhere. This may make NMP and L-proline derivatives in general interesting for further translational research in epilepsy and seizure-related diseases.
